# Oropharyngeal Cancer Survival: A Population-Based Study of Patients Diagnosed between 1978 and 2002

**DOI:** 10.5402/2012/207263

**Published:** 2012-08-08

**Authors:** Dyego Leandro Bezerra de Souza, María Milagros Bernal, Javier Jerez Roig, Maria Paula Curado

**Affiliations:** ^1^Departamento de Saúde Coletiva, Universidade Federal do Rio Grande do Norte, 59010-000 Natal, Brazil; ^2^Departamento de Microbiología, Medicina Preventiva y Salud Pública, Universidad de Zaragoza, 50005 Zaragoza, Spain; ^3^Servicio de Rehabilitación, Hospital Can Misses, 07800 Ibiza, Spain; ^4^Department of Epidemiology, International Prevention Research Institute (IPRI), 69006 Lyon, France; ^5^Goiania Population-Based Cancer Registry, 74605-070 Goias, Brazil

## Abstract

*Objective*. This paper aims at studying oropharyngeal cancer survival from the Population-Based Cancer Registry of Zaragoza, Spain, for the 1978–2002 period. *Methods*. The survival rates were calculated by the Kaplan-Meier method, and the automated calculation method of the Catalan Institute of Oncology was utilized to obtain the relative survival. *Results*. The oropharyngeal cancer survival rate was 61.3% in the first year and 33.9% in the fifth year. One-year relative survival was 62.2% (CI 95%: 57.4–67.4), and five-year relative survival was 36.6% (CI 95%: 31.8–42.1). Comparison of survival rates by sex revealed statistically significant differences (*P* value = 0.017) with better survival in women. There were no differences when comparing the three age groups and the three studied time periods 1978–1986, 1987–1994, and 1995–2002. *Conclusions*. The data suggests that there were no significant changes in oropharyngeal cancer survival in the province of Zaragoza throughout the years.

## 1. Introduction

Oropharyngeal cancer represents approximately 2% of all incident cancer cases worldwide [1]. The principal risk factors are tobacco smoking and alcohol consumption, and the association of both factors is highly synergistic [2]. Genetic factors, dietary habits, human papillomavirus (HPV) infection, consumption of hot mate (traditional South American infused drink), and poor oral health are also risk factors [3–5]. As lifestyles and human behaviour are directly affected by these factors, social inequalities are related to increases in risk [6]. Squamous cell carcinomas represent 90% of oropharyngeal cancer tumours [7].

The most recent oropharyngeal cancer incidence data of Spain reveals that the country is situated amongst those that present elevated incidence in men, with rates of 3.6 cases per 100,000 inhabitants/year (CI 95% = 3.4–3.8), along with France, Slovenia, Switzerland, and Germany. For women, Spain is amongst the countries with lowest incidences, with a rate of 0.2 cases per 100,000 inhabitants/year (CI 95% = 0.2-0.3). The ratio between genders was 14.5 [8].

Oropharyngeal cancer is rarely described separately from others head and neck tumours. The anatomical proximity between the oral cavity and the oropharynx, along with the fact that the risk factors are the same, favours that both types of cancers are incorrectly analysed in a combined manner. Besides, some publications analyse these locations in conjunction with other types of cancers that present different behavior and risk factors, such as nasopharynx and hypopharynx cancers, and denominate the entire group as oral cavity and pharynx cancer [9]. Other publications separate between oral cavity and oropharynx but include base of tongue cancers in the oral cavity classification, when the base of tongue should be analysed as a sublocation of the oropharynx [8].

All these problems in the classification of oral cavity and oropharynx cancers hinder a more detailed analysis of the changes in epidemiological profiles, of the effect of treatment, and consequently, of the survival rates of both types of cancers. Recently, studies have proved that some risk factors are more related to specific locations, such as HPV infection and oropharynx locations, and tobacco and alcohol with oral cavity [10]. Separate analysis, therefore, can reveal different data for oropharyngeal cancer [11].

The objective of this study is to determine oropharyngeal cancer survival from the Population-Based Cancer Registry (PBCR) of Zaragoza, Spain. Cancer survival analysis from population-based data constitutes a valuable tool for the evaluation of welfare services offered and allows for the orientation towards diagnose and treatment strategies.

## 2. Materials and Methods

The population studied was diagnosed with oropharyngeal cancer in the province of Zaragoza, Spain, between January 1, 1978 and December 31, 2002. Data was actively collected, analysed, and monitored by the PBCR of Zaragoza until December 31, 2007. The following locations were included: base of tongue, lingual tonsils, soft palate, uvula, tonsils, and oropharynx (ICD codes [12]: C01.9, C02.4, C05.1, C05.2, C09.0, C09.1, C09.8, C09.9, C10.0–C10.4, C10.8 y C10.9).

The difficulty in establishing survival rates at a population level lies firstly in obtaining reliable data on cancer incidence for the population, and secondly, in carrying out the monitoring with accuracy and integrity. Monitoring of cases was carried out by the Cancer Registry itself through the mortality registry of the Aragon Government, which is administered by the Statistical Institute of Aragon. Database crossing and processing was initially carried out manually and gradually become computerized. All new cases registered as major salivary gland cancer were included in the analysis, except for the cases diagnosed through death certificates. Data were obtained directly from the Cancer Registries. The PBCR data of Zaragoza was the first Spanish data to be published in *Cancer Incidence in Five Continents*, appearing in this publication since volume III. A set of quality indicators evaluates the data prior to publication in CI5; the registry data of Zaragoza fulfils the established quality standards and presents excellent quality [13, 14].

Calculation of the survival rate was carried out by the Kaplan-Meier method, and relative survival was calculated through the webpage of the Catalan Institute of Oncology (CIO). The relative survival rate is defined as the relationship between the observed survival and the expected survival in a group of healthy people of similar age and gender. In practice, the survival of people without cancer is difficult to predict, and for this reason the general mortality rate of the population is used. The CIO webpage uses the Hakulinen method to calculate the relative survival of the database sent by the user and is based on the mortality tables of the Mortality Registries of the autonomous communities and provinces of Spain [15, 16].

When the number of risk patients was lower than 15, these results were not considered in the analysis, as the final estimations were unstable [17]. Survival was studied by gender, age groups (40–64 and over 65 years of age) and location (base of tongue, tonsils, soft palate, and oropharynx). In order to study the dynamics of survival, the data was stratified in three study periods (1978–1986, 1987–1994, and 1995–2002), and the survival indicators were compared. The effect of each prognostic factor (sex, age group, location, and time period) on the survival rates was evaluated by the Log-rank test. The Log-rank test is a statistical hypothesis contrast test, used to compare two or more survival curves, and the null hypothesis is that the survival of the groups under comparison is the same.

## 3. Results

The distribution of cases, number of deaths, and percentage of censored cases according to sex, age, and period are presented in Table 1. The number of incidence cases included in the study was 380, after exclusion of the cases registered through death certificates. It was observed that 87.6% of cases occurred in men; the censored percentage was 26.6% (24.3% men and 42.6% women).

The observed survival after one year of diagnosis of oropharyngeal cancer was 61.3% (CI 95%: 56.4–66.2). After five years, survival was 33.9% (CI 95%: 29.2–38.6). One-year relative survival was 61.9% (CI 95%: 56.8–67.5) in men and 64.3% (CI 95%: 51.9–70.8) in women; five-year relative survival was 34.2% (CI 95%: 29.2–40.1) in men and 53.3% (CI 95%: 40.3–70.5) in women (Table 2).

Comparison of survival rates by sex revealed statistically significant differences (*P* value = 0.017) with better survival in women. There were no differences when comparing the three age groups (*P* value = 0.61), the locations (*P* value = 0.25), and the three studied time periods (*P* value = 0.17).

## 4. Discussion

The continuous increase in the number of cancer survivors along with population ageing in Spain, with a consequent increase in the number of cases, translate into new challenges that have to be overcome by the health care system [18]. Nowadays in Spain, more than 50% of cancer patients are alive after five years of diagnosis and the trend is to increase this percentage [19].

As a consequence of improvements in diagnose tests and treatments, an increase in cancer survivors is expected, and this situation creates new demands in welfare services, which must consider such complexity for patient monitoring. It is estimated that in 2015 in Spain, 136,002 new cancer cases are diagnosed in men and 85,818 in women [1]. Actually, knowledge on the health situation of long-term survivors is still limited. It is known that the adoption of healthy lifestyles, selfcare attitude, and sociocultural and psychological aspects play an important role in the duration and life quality of patients [20, 21].

The EUROCARE studies are the most important survival data used for comparison purposes against this study. EUROCARE is a multicentric project that gathers population-based cancer registries for European countries. Initially 11 countries participated, and currently 23 countries participate in its fourth edition. Survival data from this study shows that the highest survival rates are in Western Europe, independently from the period studied [22].

For the 1990–1994 period, average survival for Europe was 28.7% (CI 95% = 26.0–31.5) in men and 43.5% in women (CI 95% = 37.0–51.0), after five years. The highest survival rates occurred in Sweden (46.6%, CI 95% = 40.7–53.4 in men and 56.2%, CI 95% = 47.5–66.4 in women), and the lowest rates were obtained in the Czech Republic, Estonia, and Slovakia. Spanish data is within the European average, with a 29% five-year survival rate for men (CI 95% = 24.0–35.0) and 44.1% (CI 95% = 27.7–70.3) for women. Spanish data originates from Grenada, Majorca, Murcia, Navarre, and Tarragona. In Zaragoza, the data encountered for the 1987–1994 period corroborates these findings, with a survival percentage of 30.4% (CI 95% = 23.3–39.6) after five years [23].

For the 1995–1999 period, the average for all European countries reveals that 37.6% of men and 49.6% of women survived after five years of diagnosis. Once more, the highest survival rates were registered in Western Europe. Sweden and Denmark presented the highest survival rates, with five-year survival rates of 45.3% (CI 95% = 40.2–51.1) for men and 44.1% (CI 95% = 37.7–51.7) for women. Survival rates for Spain were 33.1% (CI 95% = 26.0–42.0), and the lowest survival rates were found in Portugal and Northern Ireland [22].

The economic situation of countries and the amount of resources destined to welfare assistance are the main reasons for such a variation in survival rates and explain the lowest survival rates found in Eastern Europe. Nevertheless, low survival rates in countries such as Northern Ireland or other regions of the United Kingdom could also be associated with other aspects, such as the structure of the health care system, comorbidity, and risk factor patterns [24, 25].

Regarding gender, the results for Zaragoza indicate that women present higher survival rates and most part of the studies found results in the same direction. The reasons for these higher survival rates in women could be associated with a biological superiority of women in response to illness and treatment, or a higher awareness in women concerning their bodies, and consequently, a higher percentage of early-state diagnosis [24, 26].

 Higher survival rates in women were also found by another study, which investigated 89 population-based cancer registries in several European countries for the head and neck regions. However, data analysis was restricted to the period 2000–2002. Oropharyngeal cancer results demonstrate highest relative survival rates in women (47.67%) than in men (37.67%), in younger ages and in Northern Europe [27].

 The fact that along with an increase in age there is a significant decrease in survival has been published in all EUROCARE studies. The reasons are comorbidity and the therapeutics used in elder patients, where many times surgical treatments are not indicated [22, 23]. The results for the population of Zaragoza did not show significant differences when comparing survival rates in the age groups of 40–64 and over 65 years of age. The low number of diagnosed cases in patients with 39 years of age or less prevented the survival rate analysis for this group.

Regarding location, it was observed that in Zaragoza the highest survival rate was found for cancers located in the tonsils and the lowest, for those located in the tongue. A recent study carried out with data from the Surveillance, Epidemiology End Results (SEER), a program of the United States National Cancer Institute, compared cancers located in the oropharynx with those in the oral cavity. The results revealed a higher survival rate for cancers located in the oral portion of the tongue than for cancers located in the tonsils and base of tongue. This division was adopted to differentiate those locations that supposedly present a higher risk for the development of cancers associated with HPV, such as oropharyngeal cancer, from those situated in the oral cavity [28].

When comparing the three studied period, no statistically significant differences were found in the survival rates for oropharyngeal cancer in Zaragoza. Spanish results published in international studies do not demonstrate significant improvement in survival rates [22, 23].

An investigation using Canadian Cancer Registry data has studies changes in survival rates for oropharyngeal and head and neck cancers for patients diagnosed between 1992 and 2001, totaling 10,860 cases in men and 4002 cases in women. The results revealed significant improvements only in men, with an increase of 13.5% in the survival rates after five years [29].

The data presented herein suggest that Zaragoza presents survival rates similar to other Spanish registries already published. Nevertheless, no statistically significant changes have been identified when dividing and comparing the total study period of 25 years in three study periods. These results must be interpreted with caution because it is difficult to followup cases in such a long span study. Although there are limitations, the authors consider that that survival studies using data from population-based cancer registries must be carried out and published, as they allow for the evaluation of the results obtained when treating the illness in the studies population. Investigations such as the one presented herein can be the first step for the development of more effective treatment, prevention and control programs for cancer, improvement in the follow-up process of patients and future research.

Advances in the treatment of cancer and the increase in the number of survivors call for a progressively wider monitoring of this part of population. Meanwhile, there is still much to know about these patients. Studies on cancer survival need to increase follow-up time and broaden perspectives, with the objective of knowing the physical, psychological, and social aspects associated with this illness [18, 30].

## Figures and Tables

**Table 1:** Distribution of cases, the number of deaths according sex, age, and period.

	Number of persons at risk	%	Number of deaths
Sex			
Male	333	87.6	252
Female	47	12.4	27
Total	380	100	279
Age			
0–39	14	3.6	9
40–64	262	68.9	195
65+	104	27.3	75
Location			
Base of tongue	143	37.6	115
Tonsil	119	31.4	80
Soft palate	34	8.9	25
Oropharynx	84	22.1	59
Period			
1978–1986	100	26.3	67
1987–1994	139	36.5	112
1995–2002	141	37.1	100

**Table 2:** Observed and relative survival after 1, 3, and 5 years of diagnosis of oropharynx cancer.

Survival	Observed						Relative					
	1 year		3 years		5 years		1 year		3 years		5 years	
	%	CI 95%	%	CI 95%	%	CI 95%	%	CI 95%	%	CI 95%	%	CI 95%
Sex												
Men	61	55.9–66.0	37.5	32.2–42.7	31.5	26.6–36.4	61.9	56.8–67.5	39.2	34.1–45.0	34.2	29.2–40.1
Women	63.8	50.2–77.3	57.4	43.2–71.5	51.1	36.8–65.4	64.3	51.9–79.8	58.6	45.8–75.0	53.3	40.3–70.5

Total	61.3	56.4–66.2	40	35.1–44.9	33.9	29.2–38.6	62.2	57.4–67.4	41.6	36.8–47.1	36.6	31.8–42.1

Age												
40–64	64.1	58.4–69.7	40.5	34.6–46.3	34.4	28.7–40.0	64.6	59.0–70.8	41.4	35.8–48.0	36	30.5–42.6
65+	50	40.4–59.6	36.5	27.2–45.7	29.8	20.9–38.6	52	42.9–63.0	40.8	31.7–52.5	37.1	27.6–49.8
Location												
Base of tongue	58	50.0–66.0	31.5	23.8–39.1	28	20.5–35.4	59.1	51.4–67.9	33.2	26.1–42.3	31.2	24.0–40.6
Tonsil	67.2	58.7–75.6	46.2	37.1–55.2	40.3	31.5–49.1	63.1	54.9–72.6	48	39.6–58.3	43.5	35.0–54.1
Soft palate	70.6	55.3–85.8	47.1	30.2–63.9	32.4	16.7–48.1	68.9	54.6–86.9	49.1	34.4–70.2	34.4	21.2–56.0
Oropharynx	64.3	54.1–74.4	44	33.4–54.6	35.7	25.5–45.9	64.1	54.4–75.5	44.5	34.8–57.0	38.3	28.8–51.0
Period												
1978–1986	60	50.4–69.6	48	38.2–57.8	42	32.4–51.6	61.1	52.0–71.7	50.1	40.9–61.5	45.6	36.2–57.4
1987–1994	62.9	54.8–70.9	32.4	24.5–40.2	28.1	20.6–35.5	60.6	52.9–69.5	33.8	26.6–42.9	30.4	23.3–39.6
1995–2002	65.2	57.3–73.0	41.8	33.5–50.0	34	26.1–41.8	64.6	57.0–73.1	43.3	35.6–52.6	36.5	29.0–45.9

## References

[1] GLOBOCAN 2008 Cancer incidence and mortality worldwide. http://globocan.iarc.fr/.

[2] Marron M, Boffetta P, Zhang Z-F (2010). Cessation of alcohol drinking, tobacco smoking and the reversal of head and neck cancer risk. *International Journal of Epidemiology*.

[3] Andrews E, Seaman WT, Webster-Cyriaque J (2009). Oropharyngeal carcinoma in non-smokers and non-drinkers: a role for HPV. *Oral Oncology*.

[4] Lissowska J, Pilarska A, Pilarski P (2003). Smoking, alcohol, diet, dentition and sexual practices in the epidemiology of oral cancer in Poland. *European Journal of Cancer Prevention*.

[5] Lucenteforte E, Garavello W, Bosetti C, La Vecchia C (2009). Dietary factors and oral and pharyngeal cancer risk. *Oral Oncology*.

[6] Conway DI, Petticrew M, Marlborough H, Berthiller J, Hashibe M, Macpherson LMD (2008). Socioeconomic inequalities and oral cancer risk: as systematic review and meta-analysis of case-control studies. *International Journal of Cancer*.

[7] Curado MP, Hashibe M (2009). Recent changes in the epidemiology of head and neck cancer. *Current Opinion in Oncology*.

[8] De Camargo Cancela M, De Souza DLB, Curado MP (2012). International incidence of oropharyngeal cancer: a population-based study. *Oral Oncology*.

[9] Moore SR, Pierce AM, Wilson DF (2000). ‘Oral cancer’—the terminology dilemma. *Oral Diseases*.

[10] Gillison ML (2009). HPV and prognosis for patients with oropharynx cancer. *European Journal of Cancer*.

[11] De Souza DLB, De Camargo Cancela M, Perez MMB, Curado MP (2012). Trends in the incidence of oral cavity and oropharyngeal cancers in Spain. *Head and Neck*.

[12] Fritz A, Percy C, Jack A, Shanmugaratnam K, Sobin L, Parkin DM (2000). *International Classification of Diseases for Oncology (ICD-O)*.

[13] Navarro C, Martos C, Ardanaz E (2010). Population-based cancer registries in Spain and their role in cancer control. *Annals of Oncology*.

[14] Ferlay J, Parkin DM, Curado MP, Bray F, Edwards B, Shin HR (2010). *Cancer Incidence in Five Continents, Volumes I To IX*.

[15] Clèries R, Ribes J, Moreno V (2006). Relative survival computation. Comparison of methods for estimating expected survival. *Gaceta Sanitaria*.

[16] Clèries R, Valls J, Esteban L (2007). WAERS: an application for web-assisted estimation of relative survival. *Informatics for Health and Social Care*.

[17] Jensen OM, Parkin DM, MacLennan R, Muir CS, Skeet RG (1991). *Cancer Registration: Principles and Methods*.

[18] Ferro T, Borràs JM (2011). The growing snowball in health services: long-term cancer survivors. *Gaceta Sanitaria*.

[19] Francisci S, Capocaccia R, Grande E (2009). The cure of cancer: a European perspective. *European Journal of Cancer*.

[20] Demark-Wahnefried W, Aziz NM, Rowland JH, Pinto BM (2005). Riding the crest of the teachable moment: promoting long-term health after the diagnosis of cancer. *Journal of Clinical Oncology*.

[21] Gilliam MB, Madan-Swain A, Whelan K, Tucker DC, Demark-Wahnefried W, Schwebel DC (2012). Social, demographic, and medical influences on physical activity in child and adolescent cancer survivors. *Journal of Pediatric Psychology*.

[22] Sant M, Allemani C, Santaquilani M, Knijn A, Marchesi F, Capocaccia R (2009). EUROCARE-4. Survival of cancer patients diagnosed in 1995–1999. Results and commentary. *European Journal of Cancer*.

[23] Sant M, Aareleid T, Berrino F (2003). EUROCARE-3: survival of cancer patients diagnosed 1990–1994—results and commentary. *Annals of Oncology*.

[24] Micheli A, Ciampichini R, Oberaigner W (2009). The advantage of women in cancer survival: an analysis of EUROCARE-4 data. *European Journal of Cancer*.

[25] Brenner H, Francisci S, de Angelis R (2009). Long-term survival expectations of cancer patients in Europe in 2000-2002. *European Journal of Cancer*.

[26] Micheli A, Mariotto A, Giorgi Rossi A, Gatta G, Muti P (1998). The prognostic role of gender in survival of adult cancer patients. *European Journal of Cancer*.

[27] Van Dijk BAC, Gatta G, Capocaccia R, Pierannunzio D, Strojan P, Licitra L (2012). Rare cancers of the head and neck area in Europe. *European Journal of Cancer*.

[28] Saba NF, Goodman M, Ward K (2011). Gender and ethnic disparities in incidence and survival of squamous cell carcinoma of the oral tongue, base of tongue, and tonsils: a surveillance, epidemiology and end results program-based analysis. *Oncology*.

[29] Johnson-Obaseki S, McDonald JT, Corsten M, Rourke R (2012). Head and neck cancer in canada: trends 1992 to 2007. *Otolaryngology—Head and Neck Surgery*.

[30] Feliu J, Virizuela JA (2011). Follow-up of cancer survivors: a shared responsibility. *Medicina Clinica*.

